# Thymus and Activation-regulated Chemokine as a Biomarker for IgG4-related Disease

**DOI:** 10.1038/s41598-020-62941-9

**Published:** 2020-04-07

**Authors:** Masataka Umeda, Tomoki Origuchi, Shin-ya Kawashiri, Tomohiro Koga, Kunihiro Ichinose, Kaori Furukawa, Tomohito Sato, Sousuke Tsuji, Yushiro Endo, Ayuko Takatani, Toshimasa Shimizu, Shoichi Fukui, Naoki Iwamoto, Takashi Igawa, Mami Tamai, Hideki Nakamura, Atsushi Kawakami

**Affiliations:** 1 Department of Immunology and Rheumatology, Nagasaki University Graduate School of Biomedical Sciences, Nagasaki, Japan; 2 Medical Education Development Center, Nagasaki University Hospital, Nagasaki, Japan; 3 Department of Locomotive Rehabilitation, Graduate School of Biomedical Sciences, Nagasaki University, Nagasaki, Japan; 4 Departments of Community Medicine, Nagasaki University Graduate School of Biomedical Sciences, Nagasaki, Japan; 5 Center for Bioinformatics and Molecular Medicine, Nagasaki University Graduate School of Biomedical Sciences, Nagasaki, Japan

**Keywords:** Immunology, Diseases, Rheumatology

## Abstract

High serum concentrations of thymus and activation-regulated chemokine (TARC) are observed in allergic diseases such as atopic dermatitis and bronchial asthma. Frequent allergic symptoms have been reported in patients with IgG4-related disease (IgG4-RD). We investigated the pathogenic role of TARC as a biomarker in IgG4-RD patients. We evaluated the serum concentrations of TARC from 29 IgG4-RD patients, 28 primary Sjögren syndrome (pSS) patients, and 23 healthy controls (HCs) by enzyme-linked immunosorbent assay (ELISA). We analyzed the correlations between the TARC concentrations and the subjects’ clinical parameters. To investigate the biological effect of TARC on the pathogenesis of IgG4-RD, we evaluated the *in vitro* induction of plasmablasts from IgG4-RD patients by TARC. The serum concentrations of TARC in the IgG4-RD patients were significantly higher than those of the pSS patients and HCs. The serum TARC concentration of the IgG4-RD group was positively correlated with the IgG4-RD responder index (IgG4-RD RI) score and with the number of organs involved, but it was not correlated with the serum IgG4 level or eosinophil number in the IgG4-RD patients’ peripheral blood. The patients who had lung involvement had higher serum TARC concentrations. *In vitro*, TARC clearly induced the formation of plasmablasts from the IgG4-RD patients’ peripheral blood mononuclear cells. Collectively, our data suggest that a systemic increment of TARC may contribute to the development of IgG4-RD through an aberrant induction of plasmablasts.

## Introduction

TARC (thymus and activation-regulated chemokine), also known as C-C Motif chemokine ligand 17 (CCL17), is expressed in the thymus and is produced by dendritic cells, endothelial cells, keratinocytes, and fibroblasts^1^. TARC has affinity as a ligand for the C-C chemokine receptors CCR4 and CCR8, which are predominantly expressed by Th2 cells, and TARC induces Th2-dominant inflammatory reactions^2–4^.

TARC has an important role in allergic diseases such as atopic dermatitis and bronchial asthma. High serum concentrations of TARC are observed in patients with atopic dermatitis, and its concentration is closely related to disease activity^1,5^. The measurement of serum TARC concentrations has already been clinically applied as a useful marker reflecting the disease activity of atopic dermatitis^6^.

The concentration of TARC is also reported to be elevated not only in serum but also in sputum of patients with bronchial asthma^7^. It has been reported that the bronchial epithelium of patients with bronchial asthma expresses TARC^8^. The blockade of the TARC/CCR4 axis by using anti-TARC antibodies and anti-CCR4 antibodies was reported to attenuate airway inflammation in a murine model of asthma^9,10^. In light of these reports, TARC has been suspected to be involved in the pathogenesis of allergic diseases, and TARC may thus have potential as a therapeutic target.

Immunoglobulin G4-related disease (IgG4-RD) is a relative new disease entity which is characterized by the elevation of the serum IgG4 concentration, tumor-like swelling of involved organs, and the infiltration of IgG4-positive plasma cells. Multiple organs are involved, such as the salivary and lacrimal glands, thyroid, lymph nodes, pancreas, bile duct, kidney, and lung; tissue fibrosis and sclerosis occasionally cause serious organ damage^11^.

The pathogenesis of IgG4-RD is not yet clear. Both an allergic and an autoimmune disorder have been speculated to be responsible for the pathogenesis^12^. A subset of IgG4-RD patients suffer from allergic diseases, and they also show elevated serum IgE levels and eosinophil counts^13,14^. Conversely, elevated serum IgG4 concentrations are also observed in allergic diseases^15,16^. Th2 cytokines have been thought to be responsible for the production of IgG4. Th2-related mRNA expressions such as those of interleukin (IL)-4, IL-5, IL-10, and GATA-3 were elevated on CD4^+^ T cells from patients with IgG4-RD^17^. Elevated mRNA expressions of IL-4, IL-5 and IL-10 were also observed in labial salivary glands (LSGs) of patients with IgG4-RD^18^. The numbers of Th2 cells in peripheral blood were increased in IgG4-RD patients who had concomitant allergic diseases^19^.

Based on the above-described findings, we hypothesized that TARC is involved in the pathogenesis of IgG4-RD through the activation of Th2 cytokines. In fact, an increased mRNA expression of TARC in the local site of LSGs has been reported in IgG4-RD patients^18^. However, the systemic condition and the effects of TARC in IgG4-RD are not known. We conducted the present study to determine the relationships between serum TARC and several clinical parameters of IgG4-RD patients, and we performed an *in vitro* experiment to evaluate the underlying effects of TARC on the introduction of plasmablasts.

## Results

### The serum concentration of TARC was increased in the patients with IgG4-RD

**Figure 1:**
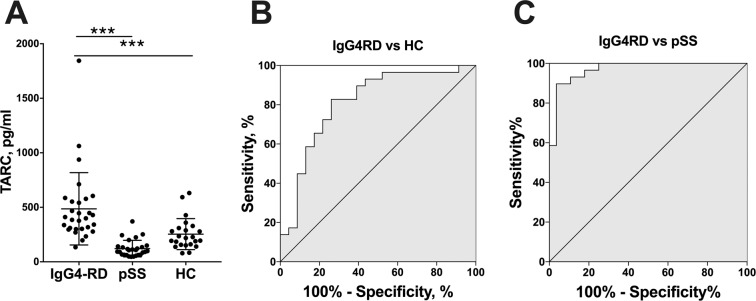
The serum concentration of TARC was elevated in the IgG4-RD patients. (**A**) The serum TARC concentrations of the IgG4-RD patients (n = 29), primary Sjögren syndrome (pSS) patients (n = 28), and healthy controls (HCs; n = 23) as analyzed by ELISA. (**B**) The receiver operator characteristic (ROC) curve for TARC distinguished the IgG4-RD patients from the HCs. (**C**) from the pSS patients. Bars: mean ± SD. ***p < 0.001 by one-way ANOVA.

The subjects’ serum concentrations of TARC were analyzed by ELISA. All of the serum samples from the IgG4-RD patients were obtained on the day before any type of immune-suppressive therapy (including glucocorticoids) was initiated. The ELISA revealed that the serum concentrations of TARC were significantly higher in the IgG4-RD group (mean 486.1 pg/ml) than those of the pSS and HC groups at mean 121.3 pg/ml and 254.2 pg/ml, respectively (Fig. 1A).

To investigate the usefulness of the serum concentration of TARC for the diagnosis of IgG4-RD, we then generated receiver operating characteristics (ROC) curves for TARC to distinguish the patients with IgG4-RD from HCs or the patients with pSS. The area under the curve (AUC) for IgG4-RD vs HCs was 0.81 and the cut-off value of TARC was 296.5 pg/dl, with relatively high sensitivity (82.8%) and specificity (73.9%) (Fig. 1B). Additionally, the AUC for IgG4-RD vs pSS was 0.97 and the cut-off value of TARC was 269.9 pg/dl, with sensitivity (89.7%) and specificity (96.4%) (Fig. 1C).

### The serum concentration of TARC was correlated with the IgG4-RI score and the number of organs involved in the IgG4-RD patients

**Figure 2:**
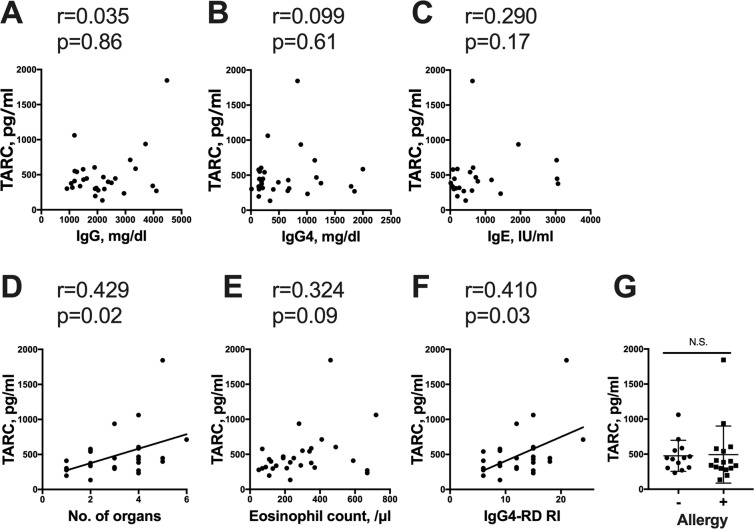
Relationships between the serum TARC concentration and clinical parameters in the IgG4-RD patients. (**A–F**) The correlations between the serum concentration of TARC and the patients’ IgG4, IgG, and IgE titers, eosinophil count, number of organs involved, and IgG4-RD responder index (IgG4-RD RI) score. The number of organs involved and the IgG4-RD RI score showed positive correlations with the serum TARC concentration. (**G**) The serum TARC concentration was not significantly different between the patients with and without allergy. All correlations were determined using Spearman’s correlation coefficient. Bars: mean ± SD. N.S.: not significant by Mann-Whitney test; n = 29, but the correlation between the serum concentration of TARC and IgE: n = 24.

To clarify the functional significance of TARC in IgG4-RD, we further examined the correlation between the serum concentration of TARC and clinical parameters in the patients with IgG4-RD including the serum IgG4, IgG, and IgE concentrations, the eosinophil count, the IgG4-RD RI score, the number of organs involved, and allergic history. Our analyses revealed that the IgG4-RD patients’ serum concentrations of TARC were positively correlated with the IgG4-RD RI score and the number of organs involved, but not correlated with the serum IgG4 level or the eosinophil number in peripheral blood (Fig. 2). No difference in the TARC concentrations was found among the IgG4-RD patients with versus without allergic symptoms.

### The serum TARC concentrations were increased in the IgG4-RD patients who had lung involvement

We analyzed the relationship between the serum TARC concentration and the organs involved. Of all of the organs shown in Table 1, the lungs were the only organ showing a significant difference in the TARC concentration between the patients with and without organ involvement. The patients group showing lung involvement had high serum TARC concentrations.

**Table 1:** The relation between TARC and organ involvement.

Affected organ	No. (%)	TARC, pg/ml		p-value
		Involved	Not involved	
Pituitary gland	2/29 (6.9%)	512.1 (238.8)	484.1 (65.0)	0.37
Orbits and lacrimal glands	13/29 (44.8%)	481.5 (93.7)	489.8 (84.5)	0.35
Salivary glands	19/29 (65.5%)	524.0 (76.5)	414.1 (105.0)	1
Thyroid	2/29 (6.9%)	579.7 (23.2)	479.2 (64.8)	0.21
Lymph nodes	17/29 (58.6%)	476.9 (81.9)	499.1 (97.5)	0.44
**Lungs**	**9/29 (31.0%)**	**678.9 (103.4)**	**399.3 (69.4)**	*0.04
Aorta/large blood vessels	6/29 (20.7%)	380.6 (136.0)	513.6 (69.5)	0.37
Retroperitoneum, mediastinum and mesentery	7/29 (24.1%)	550 (126.9)	465.8 (71.6)	0.18
Pancreas	5/29 (17.2%)	628.5 (148.1)	456.4 (67.6)	0.98
Bile duct and liver	1/29 (3.4%)	196.8 (333.1)	496.4 (62.9)	0.14
Kidney	2/29 (6.9%)	1,039 (211.9)	445.1 (57.7)	0.9
Prostate	4/29 (13.8%)	813.7 (154.7)	433.7 (61.9)	0.3

Data are % positive or mean (SD). *p < 0.05 by Mann-Whitney test.

The number of organs involved positively correlated with TARC concentration. Because of that, the number of organs involved can affect the result that the high concentration of TRAC was observed in patients with lung involvement. To determine whether the number of organs involved as a predictor for lung involvement or not, we conducted the univariate analysis. However, the number of organs involved didn’t show significance as a predicting factor for lung involvement (unit odd ratio: 1.68, 95% confidence interval: 0.87–3.23). Taken together, we could not find evidence that widespread disease is a predictor of lung involvement in our cohort. Moreover, there is no significant association between lung involvement and allergic symptoms by using Fisher’s exact test (p-value:0.69).

### *In vitro*, TARC induced the formation of plasmablasts from the IgG4-RD patients

Since our data showed that the serum concentration of TARC was elevated in the patients with IgG4-RD and correlated with the IgG4-RI score and the number of organs involved, we hypothesized that a systemic increment of TARC might contribute to the development of IgG4-RD.

**Figure 3:**
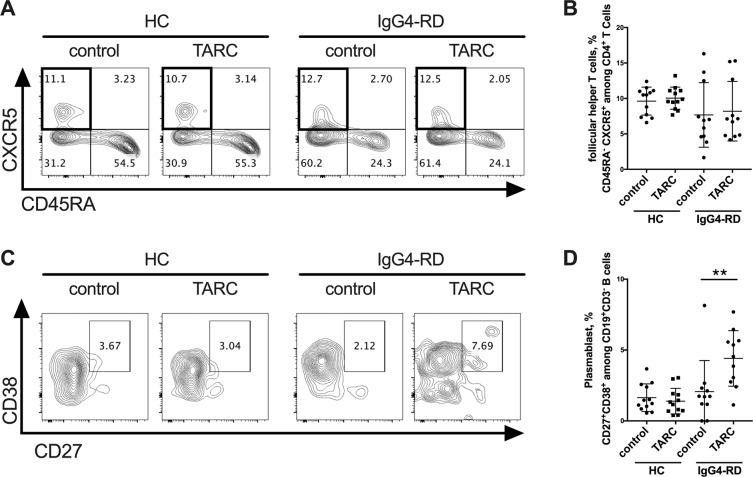
*In vitro*, TARC induced the formation of plasmablasts from IgG4-RD patients. PBMCs isolated from healthy controls and IgG4-RD patients were cultured with or without TARC (10 ng/ml) for 7 days. The incubated cells were then analyzed by flow cytometry. (**A,B**) The percentage of follicular helper-like T (Tfh) cells, identified as CD45RA^−^ CXCR5^high^ among CD4^+^ T cells was not significantly different between the cultures with and without TARC. (**C,D**) The percentage of plasmablasts, identified as CD38^+^ CD27^+^ among CD3^−^ CD19^+^ B cells from IgG4-RD patients was increased under the culture with TARC. *p < 0.05 by one-way ANOVA, healthy controls n = 12, IgG4-RD n = 11.

To examine the functions of TARC in IgG4-RD, we conducted *in vitro* experiments using PBMCs from the patients with IgG4-RD. The isolated PBMCs were incubated with or without TARC for 7 days. We then used flow cytometry to analyze the percentage of follicular helper T (Tfh) cells (CD45RA^−^ CXCR5^high^ among CD4 + T cells) and plasmablasts (CD38^+^ CD27^+^ among CD3^−^ CD19^+^ B cells) after stimulation with TARC. We observed that there was no significant difference in the induction of Tfh cells between the culture conditions with and without TARC (Fig. 3A,B). However, TARC induced the formation of plasmablasts from the patients with IgG4-RD (Fig. 3C,D).

## Discussion

To our best of our knowledge, this is the first study to report that the serum concentration of TARC was elevated in patients with IgG4-RD. The TARC concentration showed positive correlations with the number of the organs involved and the IgG4-RD RI score in the IgG4-RD patients, and the TARC concentration was higher in the patients with lung involvement. In addition, TARC *in vitro* induced the formation of plasmablasts from the patients with IgG4-RD.

The local mRNA expression of TARC was elevated in LSGs of patients with not only IgG4-RD but also SS^18^. However, our present analyses demonstrated a systemic elevation of TARC in the IgG4-RD patients but not in the pSS patients. There was a trend that TARC concentration is slightly lower in patients with pSS than in healthy controls. The previous report revealed that serum concentration of several chemokines, such as C-X-C motif chemokine ligand (CXCL)8, CCL2, CCL5, and CCL11 were significantly lower in patients with pSS than healthy controls^20^. Furthermore, the systemic manifestations of SS were reported to be low^21^, whereas IgG4-RD systemic organ involvement was reported to be frequent^13,22^. These clinical characteristics of each disease may explain the differences observed in serum TARC concentrations between IgG4-RD and pSS groups. Taking the past and present findings together, we speculate that a systemic elevation of TARC might be responsible for “the dissemination in space-like” nature of IgG4-RD which affects multiple organs.

The ROC curve generated by the ELISA results in the present study showed that TARC may be useful as a marker to distinguish individuals with IgG4-RD from healthy persons. The diagnosis of IgG4-RD presents some limitations. The definite diagnosis of IgG4-RD requires a biopsy, which is useful to detect characteristic findings such as lymphoplasmacytic infiltrations with a predominance of IgG4-positive plasma cells in the affected tissue^23^. However, a tissue biopsy is an invasive procedure, and there are some lesions that are difficult to perform a biopsy for diagnosis of IgG4-RD. It is thus desirable to establish a noninvasive way to increase the pre-test probability for diagnosis of IgG4-RD. With the use of TARC >296.5 pg/dl for distinguishing IgG4-RD from the healthy individuals in the present study population, the sensitivity was relatively high (82.8%). However, it must be kept in mind that TARC is associated with other diseases such as atopic dermatitis and bronchial asthma^1,7^.

Interestingly, we observed that the serum concentration of TARC was elevated in the patients with lung involvement. In patients with bronchial asthma, TARC has been reported to be elevated not only in serum but also in the bronchial epithelium^7,8^. The pathogenesis of IgG4-related pulmonary disease (IgG4-PD) is not yet known, but a possible relationship between asbestos exposure and IgG4-PD has been reported^24^. In light of these reports, it is possible that chronic inflammation in the respiratory system may be involved in the TARC-mediated pathogenesis of IgG4-RD.

Our analyses also showed that the serum TARC concentration in the IgG4-RD patients were not significantly different between the patients with and without allergic symptoms. TARC might be involved in the specific pathogenesis of IgG4-RD independently of the presence of allergy.

Our present *in vitro* experiments showed that TARC induced plasmablasts from PBMCs isolated from the patients with IgG4-RD. Tfh cells are reported to be necessary for B-cell differentiation including plasmablasts in the germinal center (GC)^25^, and hyperplasia of the GC with the infiltration of Tfh cells is observed in the involved organs of IgG4-RD patients^26^. Plasmablasts are thought to be play a central role in the developing pathophysiology of IgG4-RD. The number of peripheral plasmablasts in IgG4-RD patients was reported to be higher than those of healthy controls^27,28^. Another study revealed that peripheral plasmablasts show oligoclonal expansion, and their number decreases with clinical improvement after B-cell depletion therapy using rituximab^29^. Taking these findings together, we speculate that a TARC-mediated induction of plasmablasts might play an important role in the development of IgG4-RD. However, our investigation could not clarify the mechanisms by which TARC induces plasmablasts.

It is notable that the numbers of peripheral Tfh2 cells, one of the subsets of Tfh cells, are reported to expand in patients with IgG4-RD and correlate with disease activity^30^. The same study showed that Tfh2 cells *in vitro* induced the differentiation of naïve B cells into plasmablasts in IgG4-RD patients^30^. The expression of CCR4 (a receptor of TARC) was reported in IgG^+^ B cells, and TARC induced B-cell migration^31^. Although we have not analyzed Tfh2 subsets since no increase in Tfh was observed in the culture condition with TARC, we speculate that TARC may skew B cells toward to plasmablasts, directly. Of note, the percentage of plasmablasts from healthy controls was not significantly different between the cultures with and without TARC. This result suggests that plasmablasts from patients with IgG4-RD may have increased sensitively to TARC. Further research is required to elucidate the precise mechanisms underlying the induction of plasmablasts by TARC.

In conclusion, our data suggest that a systemic elevation of TARC may be involved in the development of IgG4-RD through an aberrant induction of plasmablasts. TARC could become a novel therapeutic target of IgG4-RD.

## Materials and Methods

### Study design and patients

We analyzed blood samples from individuals treated at Nagasaki University Hospital (Nagasaki, Japan) during the 8-year period from 2009 through 2017. A total of 29 patients with IgG4-RD (21 males and eight females; mean age 63.3 years, standard deviation [SD] ± 13.6 years), 28 patients with primary Sjögren syndrome (pSS) (all female; age 57.0 ± 13.3 years), and 23 healthy controls (HCs) (five males and 18 females; age 31.7 ± 9.0 years) were included. There is no significant difference in the disease duration between IgG4-RD patient group and pSS patient group (mean 54.4 ± 110.0 month vs 24.3 ± 52.7 month, respectively, p-value 0.22). Clinical features and laboratory test data were collected from medical records. All of the IgG4-RD patients fulfilled the comprehensive diagnostic criteria for IgG4-RD (2011)^23^.

Organ involvement was determined by a review of the patient’s history, physical examination findings, imaging results, laboratory studies, and tissue biopsies. Imaging included ultrasound, computed radiography, computed tomography (CT), magnetic resonance imaging, gallium scintigraphy, and positron emission tomography/CT. The demographic characteristics of patients with IgG4-RD are summarized in Table 2. The IgG4-RD responder index (IgG4-RD RI) score was obtained as described^32^. Rhinosinusitis, bronchial asthma, and drug-allergy were defined as allergic symptoms^13^.

**Table 2:** Demographics and characteristics of the 29 patients with IgG4-RD.

Males:females	21:8
Age, yrs	63.3 (13.6)
WBC count, /μL	6,311 (1614)
Eosinophil count, /μL	294.3 (200.6)
IgG, mg/dl	2,301 (995.2)
IgG4, mg/dl	606.2 (568.1)
IgE, IU/ml, n = 24	826.7 (977.3)
Allergic symptoms	13 (44.8%)
No. of organs involved	3.2 (1.4)

Values are the number (%) of patients or the mean (SD). IgG4-RD: IgG4-related disease.

All of the pSS patients fulfilled the 2016 American College of Rheumatology/European League Against Rheumatism Classification Criteria for Primary Sjögren’s Syndrome^33^.

### Ethics approval and consent to participate

This study was performed in accordance with the Declaration of Helsinki and all of the patients and HCs gave their informed consent to participate in the study and have their data published. The study was approved by the Institutional Review Board of Nagasaki University (Approval no. 15012692).

### Enzyme-linked immunosorbent assay (ELISA)

The TARC concentration in serum from the human blood samples was measured by a Human TARC enzyme-linked immunosorbent assay (ELISA) kit (R&D Systems, Minneapolis, MN). Blood samples were centrifuged within 30 min at 1500 rpm at 4 °C for 5 min, and the liquid phase of the sera was stored at −80 °C until use. Each assay was performed in triplicate independently.

### Flow cytometry

We isolated peripheral blood mononuclear cells (PBMCs) from the blood samples using density-gradient centrifugation on Sepmate and RosetteSep DM-L Density Medium (Stemcell Technologies, Grenoble, France). Isolated PBMCs were stained for flow cytometry with antibodies (Abs) against CD3 (SK7; BD Biosciences, San Diego, CA), CD4 (SK3; BD Biosciences), CD45RA (HI100; Biolegend, San Diego, CA), CXCR5 (J252D4; Biolegend), CD19 (HIB19; Biolegend), CD27 (O323; Biolegend), or CD38 (HIT2; Biolegend) for 30 min at 4 °C. Stained cells were analyzed by multiparameter flow cytometry (FACS Verse, BD Biosciences) using FlowJo ver. 10 software (BD Biosciences).

### Analysis of the effects of TARC on PBMCs

PBMCs were isolated using density-gradient centrifugation on Sepmate and RosetteSep DM-L Density Medium. The isolated PBMCs (4.0 × 10^5^) in RPMI 1640 medium with 10% fetal bovine serum (HyClone, Logan, UT) plus penicillin/streptomycin were incubated for up to 7 days at 37 °C (in 5% CO_2_) in 48-well round-bottomed plates with or without human recombinant TARC (10 ng/ml, Novus Biologicals, Littleton, CO). After stimulation with TARC, the PBMCs were harvested and stained for flow cytometry. The resulting data are representative of four independent experiments.

### Statistical analyses

Statistical significance was determined by Mann-Whitney test for two groups or one-way analysis of variance (ANOVA) for more than two groups. Spearman’s correlation coefficient with two-tailed p-values was determined in the analysis of correlations. A receiver operator characteristic (ROC) curve was described to distinguish IgG4-RD from HC by the levels of TARC. Fisher’s exact test was used to compare categorical variables. To determine the number of organ involvement as a predictor for lung involvement, univariate analysis was conducted by Wilcoxon’s test. All statistical analyses were performed in GraphPad Prism ver. 7.0 (GraphPad Software, San Diego, CA) or JMP Pro 14 software (SAS Institute, Cary, NC). P-values <0.05 were considered significant (***p < 0.001, **P < 0.01, *P < 0.05).
